# Ferrihydrite Addition Activated *Geobacteraceae*, the Most Abundant Iron-reducing Diazotrophs, and Suppressed Methanogenesis by Heterogeneous Methanogens in Xylan-amended Paddy Soil Microcosms

**DOI:** 10.1264/jsme2.ME24028

**Published:** 2024-09-12

**Authors:** Yoko Masuda, Mitsutaka Chihara, Keishi Senoo

**Affiliations:** 1 Department of Applied Biological Chemistry, Graduate School of Agricultural and Life Sciences, The University of Tokyo, 1-1-1 Yayoi, Bunkyo-ku, Tokyo 113-8657, Japan; 2 Collaborative Research Institute for Innovative Microbiology, The University of Tokyo, 1-1-1 Yayoi, Bunkyo-city, Tokyo 113-8657, Japan

**Keywords:** paddy field soil, methanogenesis, ferrihydrite, iron-reducing diazotrophs, metatranscriptome

## Abstract

Paddy fields are a major emission source of the greenhouse gas methane. In the present study, the addition of ferrihydrite to xylan-amended paddy soil microcosms suppressed methane emissions. PCR-based and metatranscriptomic ana­lyses revealed that the addition of ferrihydrite suppressed methanogenesis by heterogeneous methanogens and simultaneously activated *Geobacteraceae*, the most abundant iron-reducing diazotrophs. *Geobacteraceae* may preferentially metabolize xylan and/or xylan-derived carbon compounds that are utilized by methanogens. *Geomonas terrae* R111 utilized xylan as a growth substrate under liquid culture conditions. This may constitute a novel mechanism for the mitigation of methane emissions previously observed in ferric iron oxide-applied paddy field soils.

Rice paddy field soils are major emission sources of methane, the global warming potential (GWP) of which is greater than that of carbon dioxide (Ehhalt and Schmidt, 1978; Dijkstra *et al.*, 2012). The factors affecting methane emissions from paddy field soils have been extensively exami­ned, and the application of ferric iron oxide was shown to delay decreases in the soil redox potential (Eh) and suppress methane generation in extremely low-Eh environments (Jäckel and Schnell, 2000; Ali *et al.*, 2008). On the other hand, we previously reported that the application of ferric iron oxide to paddy soil enhanced the nitrogen-fixing activity of diazotrophic iron-reducing bacteria (*Geobacteraceae* and *Anaeromyxobacter*), the predominant diazotrophs in paddy soil (Masuda *et al.*, 2021; Zhang *et al.*, 2023). Acetate, a major metabolite of the decomposition of microbial rice straw (Glissmann and Conrad, 2000), is used as an electron donor and substrate for methanogenesis by acetate-utilizing methanogens (Falz *et al.*, 1999; Conrad, 2007), and is also utilized as an electron donor by iron-reducing bacteria (Sanford *et al.*, 2007; Masuda *et al.*, 2020). Therefore, methanogens and iron-reducing diazotrophs compete for straw-derived carbon compounds, such as acetate. Based on these findings, we hypothesized that when methanogenesis is suppressed by the application of ferric iron oxide, the activation of iron-reducing diazotrophs simultaneously occurs in paddy soil due to the preferential utilization of straw-derived carbon compounds by diazotrophic iron-reducing bacteria. The purpose of the present study was to test this hypothesis using soil microcosms amended with xylan/cellulose, the main components of rice straw.

Paddy soil microcosms were constructed as follows. Paddy soil was collected at an experimental paddy field in the Niigata Agricultural Research Institute. Ten grams of soil was amended with cellulose (36.8‍ ‍mg) or xylan (26.3‍ ‍mg), the major component of rice straw, ferrihydrite (50‍ ‍mg), a ferric iron oxide, was added, and the mixture was then placed in a vial (50‍ ‍mL) (Supplementary document, Fig. S1). Each vial containing soil and air was sealed with a butyl rubber stopper and plastic screw cap and incubated at 30°C for 31 days. The amount of methane that accumulated in the gas phase was measured periodically by gas chromatography equipped with a flame ionization detector. Detailed information on the materials and methods used is provided in the supplementary document. Soils without carbon (cellulose or xylan) amendments (N, without ferrihydrite and NF, with ferrihydrite) did not emit methane during the incubation period (Fig. 1). Methane emission was observed from soils amended with cellulose and xylan. In cellulose-amended soils (C and CF), no significant differences were observed in the amount of methane that accumulated between ferrihydrite-amended (CF) and non-amended (C) soils during the incubation period. Meanwhile, in xylan-amended soils (X and XF), the amount of methane that accumulated was significantly lower in ferrihydrite-amended soil (XF) than in non-amended soil (X) after Day 9. These results suggest that the addition of ferrihydrite reduced the substrates available for methanogenesis (acetate and CO_2_/H_2_) that were derived from the microbial decomposition of xylan in soil.

A metatranscriptomic ana­lysis of X and XF soils (Day 16) targeting methanogenesis-related gene (*mcrA*) transcripts revealed that the detection frequency of *mcrA* transcripts was significantly lower in XF soil than in X soil‍ ‍(Fig. 2A), which is consistent with the results showing that methanogenesis was suppressed in XF soil. The taxonomic composition of *mcrA*-transcribing archaea, *i.e.*, active methanogens, did not significantly differ between XF and X soils (Fig. 2B). Among microbes in the three most predominant genera, *Methanoregula* is a CO_2_/H_2_-utilizing methanogen, *Methanothrix* is an acetate-utilizing methanogen, and *Methanosarcina* utilizes both CO_2_/H_2_ and acetate (Huser *et al.*, 1982; Jetten *et al.*, 1992; Bräuer *et al.*, 2011; Bai *et al.*, 2020). These results suggest that the addition of ferrihydrite to xylan-amended paddy soils suppressed methanogenesis by heterogeneous methanogens, including that by CO_2_/H_2_-utilizing and acetate-utilizing methanogens, in a non-specific manner. The addition of ferrihydrite may have decreased both acetate and CO_2_/H_2_, which are substrates of methanogenesis derived from microbial xylan decomposition.

We also investigated whether the addition of ferrihydrite activated iron-reducing diazotrophs in xylan-amended soils (X and XF soils) and suppressed methanogenesis. In a qPCR-based ana­lysis targeting the nitrogenase gene (*nifD*) of iron-reducing diazotrophs, the relative abundance of the *nifD*/16S rRNA gene increased during the first half of the incubation period and was significantly higher in XF soils than in X soils on Days 9 and 21 (Fig. S2). These results indicate that iron-reducing diazotrophs grew in soils by utilizing xylan-derived carbon compounds as carbon and energy sources and also that their growth was stimulated by the addition of ferrihydrite. In a metatranscriptomic ana­lysis targeting *nifD* transcripts in X and XF soils (Day 16), the detection frequency of *nifD* transcripts was significantly higher in XF soil than in X soil (Fig. 3A), indicating that the‍ ‍addition of ferrihydrite activated nitrogen fixation in xylan-amended paddy soil. A taxonomic composition ana­lysis revealed that the large majority of *nifD*-transcribing microbes belonged to *Geobacteraceae*, which are iron-reducing bacteria, and their abundance was higher in XF soils than in X soils (Fig. 3B), suggesting that these microbes significantly contributed to the activation of nitrogen fixation in xylan-amended paddy soils supplemented with ferrihydrite. These results supported our hypothesis that when methanogenesis was suppressed by the addition of ferrihydrite, the activation of iron-reducing diazotrophs, mostly *Geobacteraceae*, occurred in xylan-amended paddy soil.

A possible mechanism for the simultaneous suppression of methanogenesis and activation of *Geobacteraceae* is that *Geobacteraceae* preferentially consumed acetate, the substrate of methanogenesis by acetate-utilizing methanogens. However, as described above, the addition of ferrihydrite suppressed methanogenesis by heterogeneous methanogens, including that by CO_2_/H_2_-utilizing and acetate-utilizing methanogens. Therefore, we hypothesized that members of *Geobacteraceae* may utilize carbon compounds other than acetate, *i.e.*, xylan and/or carbon compounds generated from the decomposition of xylan. A liquid culture experiment using *Geomonas terrae* R111, a representative strain of *Geobacteraceae* (Xu *et al.*, 2019), demonstrated that this strain grew by utilizing xylan as carbon and energy sources and ferric iron as an electron acceptor for respiration (Fig. S3A and B) and exhibited nitrogen-fixing activity (Fig. S3B). Consequently, the present results strongly suggest that in xylan-amended soil, the addition of ferrihydrite activated the consumption of xylan and nitrogen fixation by xylan-utilizing *Geobacteraceae*, thereby decreasing the generation of acetate and CO_2_/H_2_ from xylan decomposition and subsequently suppressing methanogenesis.

In terms of the taxonomic composition of *nifD*-transcribing microbes (Fig. 3B), *Anaeromyxobacter*, iron-reducing bacteria, were the second most dominant; however, their abundance did not significantly differ between X and XF soils. One possible reason for this is their inability to utilize xylan, unlike *Geomonas*. *Anaeromyxobacter diazotrophicus* Red267^T^, a representative strain of the diazotrophic genus *Anaeromyxobacter* (Itoh *et al.*, 2022), did not utilize xylan as its growth substrate in our cultivation study (data not shown). *Parabacteroides* and *Azospira* were significantly more abundant in XF soil than in X soil; however, their contributions to soil nitrogen fixation were small because of their low abundance among *nifD*-transcribing microbes.

As shown in Fig. 1, the addition of ferrihydrite to cellulose-amended soil did not effectively suppress methanogenesis. Unlike xylan, cellulose may not be utilized by *Geobacteraceae* in soil. *G. terrae* Red111^T^ did not utilize cellulose as a growth substrate in our culture experiment (data not shown). In addition, cellulose was not utilized by *A. diazotrophicus* Red267^T^ (data not shown). Methanogens may compete with iron-reducing bacteria for substrates (acetate and CO_2_/H_2_) derived from cellulose even in ferrihydrite-amended soil.

In summary, in xylan-amended paddy soil microcosms, the addition of ferrihydrite activated nitrogen fixation by *Geobacteraceae*, the most abundant iron-reducing diazotrophs, and suppressed methanogenesis by heterogeneous methanogens. From a mechanistic perspective, xylan itself and/or carbon compounds generated from microbial xylan decomposition appeared to be preferentially consumed by *Geobacteraceae*, leading to decreases in available acetate and CO_2_/H_2_ as substrates of methanogenesis by methanogens. This may constitute a novel mechanism for mitigating methane emissions via the application of ferric iron oxide previously observed in paddy field soils. Further studies using ^13^C-labeled carbon compounds are needed to clarify this mechanism in more detail.

## Citation

Masuda, Y., Chihara, M., and Senoo, K. (2024) Ferrihydrite Addition Activated *Geobacteraceae*, the Most Abundant Iron-reducing Diazotrophs, and Suppressed Methanogenesis by Heterogeneous Methanogens in Xylan-amended Paddy Soil Microcosms. *Microbes Environ ***39**: ME24028.

https://doi.org/10.1264/jsme2.ME24028

## Supplementary Material

Supplementary Material

## Figures and Tables

**Fig. 1.**
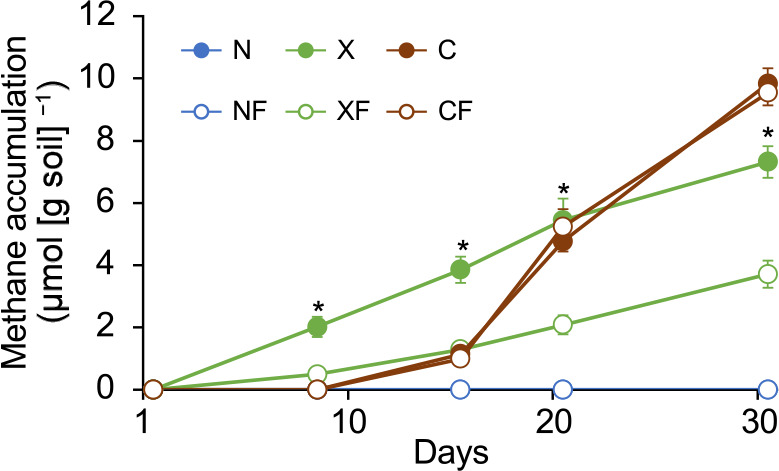
Methane accumulation in vials from control (N), ferrihydrite-amended (NF), xylan-amended (X), xylan and ferrihydrite-amended (XF), cellulose-amended (C), and cellulose and ferrihydrite-amended (CF) paddy soils (mean±SD). * indicates ferrihydrite-amended soils, in which the accumulation of methane was significantly lower than in the sample without ferrihydrite (the Mann–Whitney *U* test; *P*<0.05).

**Fig. 2.**
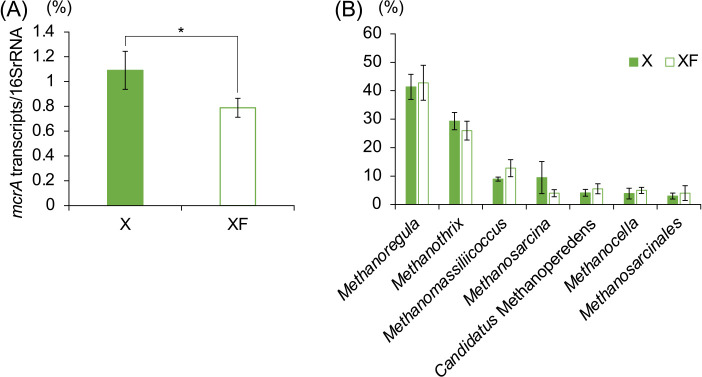
Ratio of the number of *mcrA* transcript reads to the number of rRNA transcript reads (A) and the taxonomic composition of *mcrA*-derived archaea (B) obtained by a metatranscriptomic ana­lysis of xylan-amended (X) and xylan and ferrihydrite-amended (XF) paddy soils on Day 16 (mean±SD). * indicates ferrihydrite-amended soils, in which the *mcrA* ratio was significantly lower than in soils without ferrihydrite (the Mann–Whitney *U* test; *P*<0.05).

**Fig. 3.**
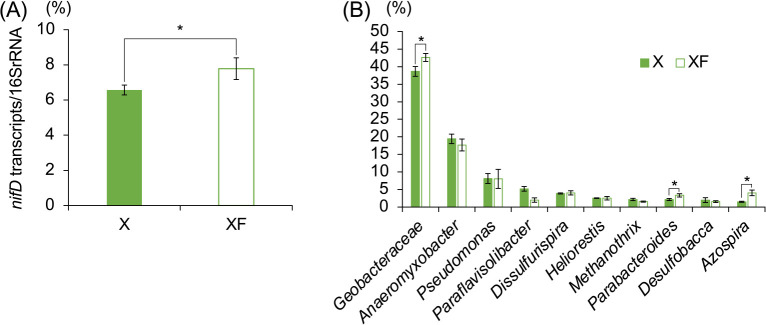
Ratio of the number of *nifD* transcript reads to the number of rRNA transcript reads (A) and the taxonomic composition of *nifD*-transcribing microbes (B) obtained by a metatranscriptomic ana­lysis of xylan-amended (X) and xylan and ferrihydrite-amended (XF) paddy soils on Day 16 (mean±SD). * indicates ferrihydrite-amended soils, in which the *nifD* ratio was significantly higher than in soils without ferrihydrite (the Mann–Whitney *U* test; *P*<0.05).
