# Supplementary material for: Ferrihydrite Addition Activated Geobacteraceae, the Most Abundant Iron-reducing Diazotrophs, and Suppressed Methanogenesis by Heterogeneous Methanogens in Xylan-amended Paddy Soil Microcosms
Source: Microbes Environ. 2024 Sep 12;39(3):ME24028. doi: 10.1264/jsme2.ME24028 (PMC11427309; doi:10.1264/jsme2.ME24028)
Supplement: Supplementary file 1 — Supplementary Material [file 39_24028_s1.pdf]

Supplementary document

Materials and Methods

The paddy soil used in this study was collected from an experimental paddy field at the Niigata Agricultural Research Institute, Nagaoka, Japan. The physicochemical properties of the soil were reported previously (Masuda et al., 2023). The soil was preincubated with 50% moisture content at 30 °C for approximately 1 week. Ten grams of the preincubated soil was placed in 50-mL glass vials (Nichiden Rika Glass, Hyogo, Japan) and amended with carbon sources (cellulose or xylan) and supplemented with ferrihydrite (Schwertmann and Cornell, 2008). The experimental groups were as follows: no carbon source amended, N; no carbon source and ferrihydrite amended, NF; xylan amended, X; xylan and ferrihydrite amended, XF; cellulose amended, C; and cellulose and ferrihydrite amended, CF (Fig. S1). Three replicates for methane measurement and DNA/RNA extraction were performed per group. The amount of each additive was as follows: 36.8 mg of cellulose, 26.3 mg of xylan, and 50 mg of ferrihydrite (Masuda et al., 2021). The amounts of xylan and cellulose were equivalent to the amount contained in 100 mg of rice straw (Yoo et al., 1989). The vials were sealed with rubber stoppers and plastic screw caps and incubated at 30 °C for 30 days.

The amount of accumulated methane was measured on Days 1, 9, 16, 21, and 30.

The methane concentration was measured by collecting 100 μL of the gas phase in a vial with a 100-μL syringe (Hamilton, NV, USA) and injecting it into a GC-2014 equipped with a fused silica column (Rt-U-Bond PLOT; Restek) and an FID detector (Shimazu, Kyoto, Japan).

On the days of the methane measurement, soil samples were collected from vials for DNA and RNA extraction and stored at -80 °C until subsequent use. Soil DNA and RNA from each experimental group were extracted and purified using a previously described method (Masuda et al., 2021). To obtain DNA/RNA solutions, RNase/DNase treatment was performed as described previously (Masuda et al., 2021). Complementary DNA (cDNA) was synthesized using the ReverTra Ace qPCR RT Master Mix with gDNA Remover kit (Toyobo, Osaka, Japan) according to the manufacturer's instructions.

Quantitative PCR was performed targeting the bacterial 16S rRNA gene and *nifD* gene of *Geobacteraceae*/*Anaeromyxobacter* in soil DNA samples using specific primer sets as described previously (Masuda et al., 2021).

Metatranscriptomic sequencing of X and XF soil samples on Day 16 was performed using the Illumina MiSeq platform (Illumina, San Diego, CA, USA) for a 2 × 300 paired-end (PE) configuration at Bioengineering Lab. Co. (Kanagawa, Japan). Trimmomatic v0.36 (http://www.usadellab.org) was used to trim adaptors and remove

low-quality sequences from the raw sequences. The clean reads were aligned to the SILVA LSU (23S/28S) and SILVA SSU (16S/18S) databases to remove rRNA reads using SortMeRNA v2.1b (http://bioinfo.lifl.fr/RNA/sortmerna/) software. The remaining clean reads were assembled with megahit v1.1.1-2-g02102e1(http://www.l3-bioinfo.com/products/megahit.html). Then, METAProdigal v2.6.3 was used to predict open reading frames (ORFs), and a nonredundant gene catalog was constructed using CD-HIT v.4.6.7 with a sequencing identity cutoff of 0.95. The gene catalog was searched against the KEGG database using KOfam v1.2.0 to annotate the functions of protein sequences. The sequences of the methane generation-related McrA and nitrogenase (NifD) proteins were extracted. To identify the microorganisms from which the sequences were derived, protein sequences were compared to those in the nr database in January 2024 by BLASTP (E value of $<10^{-5}$, alignment length of > 30 aa). The Mann-Whitney *U* test was used to compare the differences between groups. All metatranscriptomic sequences were deposited in the Data Bank of Japan (DDBJ; https://www.ddbj.nig.ac.jp) under the IDs: DRX519855, DRX519856, DRX519857, DRX519858, DRX519859, and DRX519860.

Representative iron-reducing diazotrophs isolated from paddy soils, namely, *Anaeromyxobacter diazotrophicus* Red267^T and *Geomonas terrae* Red111^T, were

cultured in MM medium (Masuda et al., 2020) or MFM medium (Xu et al., 2019) without NH_4Cl, respectively, under N_2/CO_2 (80:20 v/v) at 30 °C for 7 days. The electron acceptor and donor for both strains were 5 mM Fe(III)-nitrilotriacetic acid (NTA) and 1 g/L xylan, respectively. Growth was observed based on visual changes in the culture medium (red turbid to white turbid) derived from the reduction of ferric iron to ferrous iron, as described previously (Xu et al., 2019; Nevin et al., 2005). Nitrogen fixation activity was measured by the acetylene reduction assay (ARA) based on C_2H_2 reduction into C_2H_4 by nitrogenase as described previously (Masuda et al., 2020). The Fe^{2+} content of each culture medium was determined as described previously (Ishii et al., 2009).

References

Ishii, S., Yamamoto, M., Kikuchi, M., Oshima, K., Hattori, M., Otsuka, S., and Senoo, K. (2009) Microbial populations responsive to denitrification-inducing conditions in rice paddy soil, as revealed by comparative 16S rRNA gene analysis. Appl Environ Microbiol **75**: 7070-7078.

Nevin, K.P., Holmes, D.E., Woodard, T.L., Hinlein, E.S., Ostendorf, D.W., and Lovley, D.R. (2005) *Geobacter bemidjiensis* sp. nov. and *Geobacter psychrophilus* sp. nov., two novel Fe(III)-reducing subsurface isolates. Int J Syst Evol Microbiol **55**:1667–1674.

Masuda, Y., Shiratori, Y., Ohba, H., Ishida, T., Takano, R., Satoh, S., Shen, W., Gao, N., Itoh, H., and Senoo, K. (2021) Enhancement of the nitrogen-fixing activity of paddy soils owing to iron application. Soil Sci Plant Nutr **67**: 243-247.

Masuda, Y., Satoh, S., Miyamoto, R., Takano, R., Ishii, K., Ohba, H., Shiratori, S., and Senoo, K. (2023) Biological nitrogen fixation in the long-term nitrogen-fertilized and unfertilized paddy fields, with special reference to diazotrophic iron-reducing bacteria. Arc Microbiol **205**: 291.

Schwertmann, U., and Cornell, R.M. (2008) Iron oxides in the laboratory: preparation and characterization. John Wiley & Sons.

Xu, Z., Masuda, Y., Itoh, H., Ushijima, N., Shiratori, Y., and Senoo, K. (2019) *Geomonas oryzae* gen. nov., sp. nov., *Geomonas edaphica* sp. nov., *Geomonas ferrireducens* sp. nov., *Geomonas terrae* sp. nov., four ferric-reducing bacteria isolated from paddy soil, and reclassification of three species of the genus *Geobacter* as members of the genus *Geomonas* gen. nov. Front Microbiol **10**:2201.

Yoo, I.D., Kimura, M., Wada, H., and Takai, Y. (1991) Organic constituents of rice straw contributed to the biological N_2 fixation in paddy field: model experiment. Jpn J Soil Sci Plant Nutr **62**:219-225.

Fig.S1

Carbon compounds and ferrihydrite treatments of paddy soil microcosms.

Fig.S2

Relative abundance of copy numbers of *nifD* derived from *Anaeromyxobacter* and *Geobacteraceae* to 16S rRNA in xylan-amended paddy soils.

Fig.S3

(A) Growth of *Geomonas terrae* Red111^T in culture media containing xylan as the sole carbon source and ferric iron as the electron acceptor for respiration. Growth was monitored by the disappearance of the reddish-brown color of ferric iron. (B) Transitions of acetylene reduction activity and ferrous iron concentration in the culture medium of *Geomonas terrae* Red111^T.

Fig. S1

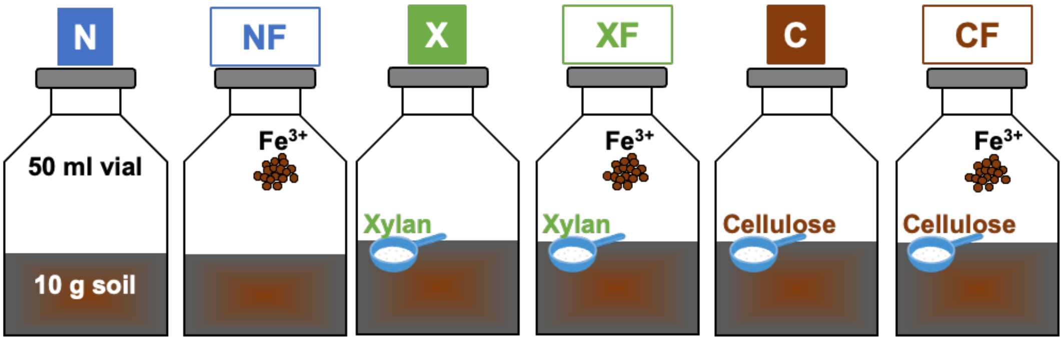
N
NF
X
XF
C
CF
50 ml vial
10 g soil
Fe3+
Fe3+
Fe3+
Xylan
Xylan
Cellulose
Cellulose

Fig. S2

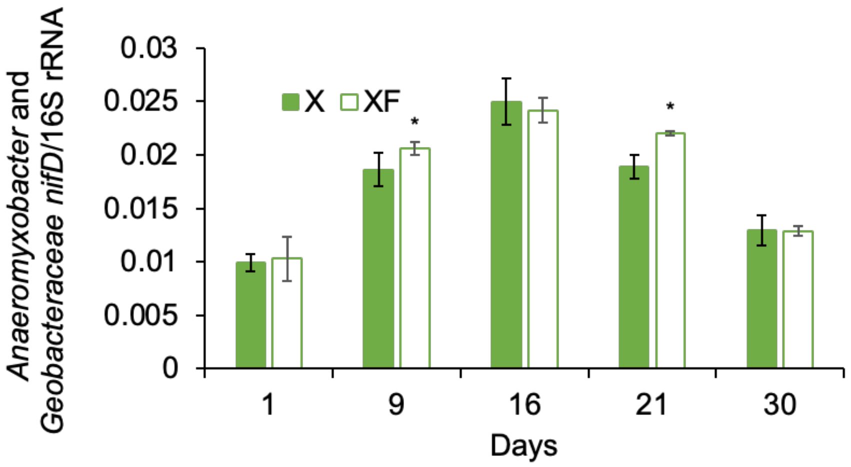
Anaeromyxobacter and Geobacteraceae nifD/16S rRNA
0.03
0.025
0.02
0.015
0.01
0.005
0
X
XF
*
*
1
9
16
21
30
Days

Fig. S3

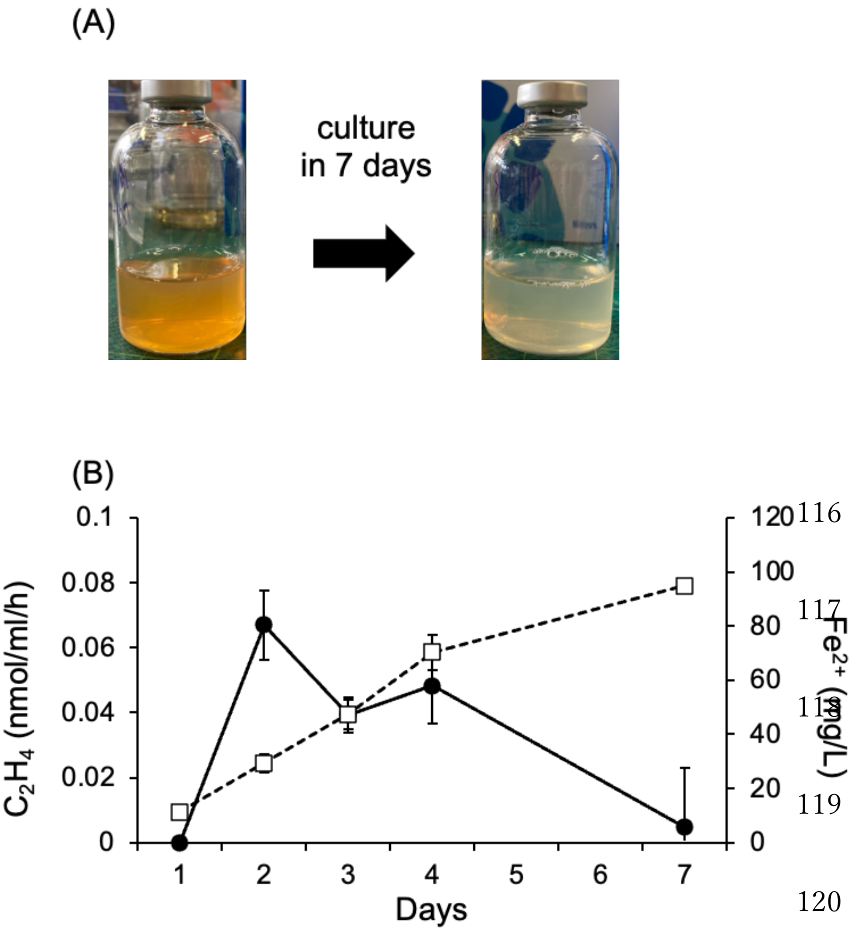
(A)
culture
in 7 days
(B)
C_2H_4 (nmol/ml/h)
0
0.02
0.04
0.06
0.08
0.1
Fe^{2+} (mg/L)
0
20
40
60
80
100
120
1
2
3
4
5
6
7
Days

Table S1 Primer sequences used in this study

Primer	Sequences (5'→3')
**16S rRNA 27F**	AGAGTTTGATCCTGGCTCAG
**16S rRNA 520R**	ACCGCGGCTGCTGGC
**A&G nifD-F**	CCTSATYGGYGACGAYATMAAC
**A&G nifD-R**	TAGTTCATGGAVCGGTARCAGT
